# Experiences of barriers and facilitators to weight-loss in a diet intervention - a qualitative study of women in Northern Sweden

**DOI:** 10.1186/1472-6874-14-59

**Published:** 2014-04-16

**Authors:** Anne Hammarström, Anncristine Fjellman Wiklund, Bernt Lindahl, Christel Larsson, Christina Ahlgren

**Affiliations:** 1 Department of Public Health and Clinical Medicine, Umeå University, Umeå, Sweden; 2 Umeå Centre for Gender Studies in Medicine, Umeå University, Umeå, Sweden; 3 Department of Community Medicine and Rehabilitation, Physiotherapy, Umeå University, Umeå, Sweden; 4 Department of Food and Nutrition, and Sport Science, University of Gothenburg, Sweden, Gothenburg, Sweden; 5 Department of Food and Nutrition, Umeå University, Umeå, Sweden

**Keywords:** Behavior change, Weight management, Obesity/overweight, Intervention programmes, Gender, Qualitative analysis, Health behavior, Women’s health/midlife

## Abstract

**Background:**

There is a lack of research about the experiences of participating in weight-reducing interventions. The aim of this study was to explore barriers and facilitators to weight-loss experienced by participants in a diet intervention for middle-aged to older women in the general population in Northern Sweden.

**Method:**

In the intervention the women were randomised to eat either a Palaeolithic-type diet or a diet according to Nordic Nutrition recommendations for 24 months. A strategic selection was made of women from the two intervention groups as well as from the drop-outs in relation to social class, civil status and age. Thematic structured interviews were performed with twelve women and analysed with qualitative content analyses.

**Results:**

The results showed that the women in the dietary intervention experienced two main barriers – struggling with self (related to difficulties in changing food habits, health problems, lack of self-control and insecurity) and struggling with implementing the diet (related to social relations and project-related difficulties) – and two main facilitators– striving for self-determination (related to having clear goals) and receiving support (from family/friends as well as from the project) – for weight-loss. There was a greater emphasis on barriers than on facilitators.

**Conclusion:**

It is important to also include drop-outs from diet interventions in order to fully understand barriers to weight-loss. A gender-relational approach can bring new insights into understanding experiences of barriers to weight-loss.

**Trial registration:**

ClinicalTrials gov NCT00692536.

## Background

Obesity is rapidly increasing around the world and is considered to be one of the most important threats to public health worldwide [1]. Therefore, both prevention of obesity and efficient programmes for weight-loss in various contexts must be in focus in health promotion. Yet although there are many treatment regimens for obesity, successful weight-loss and maintenance have been shown to be suboptimal [2,3]. Most of the dietary intervention studies have focused on evaluating the effect of the intervention regarding anthropometric or metabolic variables [2,4]. But in order to understand why a majority of the intervention programmes have shown suboptimal long-term results, and to obtain knowledge of how future programmes ought to be designed, it is crucial to analyse the experiences of the participants. Of vital importance for designing successful weight-loss programmes is an understanding of how the participants experience barriers and facilitators to losing weight. Surprisingly, despite the high amount of quantitative studies about weight reduction, qualitative research about the experiences of participating in weight-reducing interventions is sparse [4,5]. There are some qualitative studies about diet interventions aimed at patients with specific diagnoses (such as diabetes, coronary heart disease, knee problems). For example, a qualitative evaluation of an intervention was performed with high cardiovascular risk participants [6]. The study showed that the process of weight-loss was experienced as complex and challenging, due to their chronic health conditions, the pros and cons of social support (meaning that family members could either support or hinder their new diet) as well as due to the time constraints of changing the diet. Another qualitative study was performed with participants in (commercial) weight-loss programmes in a patient-based cohort study [7]. The study found that motivation was experienced as an enabler while low self-efficacy was a recurrent barrier to weight-loss attempts. A similar study, based on an intervention among primary-health care patients, identified personalized messages and social support (from professionals and others) as facilitators to successful weight management [4].

However, there is a lack of studies and thus little knowledge about the perceived experiences, attitudes and feelings of participants from the general population in dietary interventions. Therefore, the aim of this study was to explore barriers and facilitators to weight-loss experienced by participants in a diet intervention for middle-aged to older women in the general population in Northern Sweden.

## Methods

### Setting

The study was conducted in an area around a middle-sized town in Northern Sweden. The intervention was based at the hospital.

### Sampling base

Middle-aged to older women were invited by announcement to a weight-loss programme, designed as a two-year randomised clinical trial (ClinicalTrials.gov, NCT00692536). In all 70 women with a mean age of 60 years (range 49 to 71 years) with overweight/obesity (BMI > 27 kg/m^2^) were included. For practical reasons, the participants were included on three different occasions (24 participants on each occasion equally divided into two different dietary groups). After baseline measurements, half of the participants were randomised to eat a Palaeolithic-type diet (PD) aiming at an energy intake from protein, fat and carbohydrates of 30, 40 and 30 E% respectively. The other half was prescribed to eat a diet according to the Nordic Nutrition Recommendations (NNR), aiming at an energy intake from protein, fat and carbohydrates of 15, 25 and 60 E% respectively. Energy consumption was not limited and only general advice was given about physical activity. To achieve adherence to the food intake, participants in each dietary group took part in a total of 12 group sessions held by a trained study dietician (one dietician per diet) throughout the 24-months study period. The group sessions consisted of information on and cooking of the intervention diets, dietary effects on health, behavioural changes, and group discussions. The subjects were given recipes and written instructions to facilitate the preparation of meals at home. Eight group sessions (four cooking classes and four follow-up sessions) were held during the first 6 months of the intervention. Additional group meetings were held at 9, 12, 18, and 24 months. Adherence to the diet was followed by repeated food diaries and objective measurement of energy and protein intake. The intervention study started in August 2007 and was carried out until March 2010 with a drop-out of 13% after 6 months and 30% in total after 24 months. There was no significant difference between the intervention groups in drop-out rate (eight women in the PD group, 13 in the NNR group) calculated as the difference of proportions between groups (p = 0.19) [8].

Both diet groups had a significant weight-loss over time. The largest weight-loss was measured at the 12-months follow-up with −8.7 kg in the PD group and −4.4 kg in the NNR group. At the 24-months, the weight-loss was −6.2 kg and −3.7 kg, respectively. There was a significantly higher weight reduction in the PD group compared to the NNR group at the 12-months follow-up but not at the 24-months follow-up [8].

### Participants

A strategic selection of women from the whole sampling base of the intervention study was performed after its completion in Autumn 2010. A sample of fourteen women was drawn from three groups: the two diet groups (PD and NNR) as well as from the drop-outs from the intervention. We strived for getting as much variation as possible among the selected women in relation to social class/education, civil status, living in rural or urban areas and age (working age or retired). The strategic selection provided a varied group of participants in each of these three groups – including both women in working life and pensioners, those who were married and single, women living in the city as well as in the surrounding rural areas and women of varying educational background (middle-class and working-class). Two women refused to participate in our interview study and thus, twelve participated in the study. Overall, the participants were quite highly educated and all of them were Swedish-born. In total, four of the twelve women were drop-outs while eight women had fulfilled the project, with four participating in the PD and four in the NNR dietary intervention.

### Performance

Individual thematic structured interviews were performed with the aid of an interview guide with open questions regarding barriers and facilitators to weight reduction. Example of questions for this paper was: Please tell me about barriers/facilitators to achieve your goal of weight-loss. What have you done in order to overcome the barriers? Did you experience disadvantages with the new diet. If yes, which? Please tell me about the prerequisites for achieving your weight goal. What has made you succeed/not succeed in reaching your weight goal? Minor adjustments were made to the questions between interviews so that information provided in one interview could be taken into consideration in a subsequent interview. A sociologist interviewed ten of the women, while two of the authors (CA, AFW) interviewed the other two women. We performed the interviews at our workplace, in the home or at the workplace of the participants. The interviews lasted about one hour each, and were tape-recorded and transcribed verbatim.

The interviewer and all authors except BL and CL were never involved in the intervention study.

### Data analysis

The analyses were performed according to qualitative content analyses [9]. The analysis started with a naïve reading to get an understanding of the content of the interviews after which the open coding was performed. During all steps of the analysis two or three of the authors (AFW, AH, CA) coded each interview independently, followed by a mutual comparison and a final negotiated outcome between all authors. In this process, we found only minor disagreements between us and, when that occurred, we went back to the original text and discussed the coding until agreement was reached. This process of methodological triangulation was performed in order to increase the trustworthiness of the data and its interpretation [10].

The data analysis was performed in the following way. First, sentences describing obstacles and facilitators were extracted from the text as meaning units (i.e., words or sentences that convey the same central meaning). Thereafter, the meaning units were condensed and abstracted into codes. Examples of meaning units and codes are given in Table 1.

**Table 1:** Examples of meaning units and codes

**Meaning units**	**Codes**
Difficult to do without wine	Longing for certain food
Difficult to do without cheese	
Difficult to do without sweets	
Difficult to do without bread	
Difficult to do without potatoes	
Genetically determined	Cannot control weight
Need for high self-control	
Cannot control herself	
In need of wellness centre	
I eat too much	
My body is too effective, takes up too much nutriment	
My weight just increases	
The bread just goes down	

The codes were then interpreted and compared regarding differences and similarities for the two content areas – barriers and facilitators to weight-loss. After that the codes were brought together on a more abstract level into preliminary subcategories which were then sorted and abstracted into preliminary subcategories and categories. Finally, these preliminary subcategories and categories were discussed, reflected on and condensed into final subcategories and categories. A category refers mainly to a descriptive level of content and answers the question “What?” and can thus be seen as an expression of the manifest content of the text. A category often includes a number of subcategories or sub-subcategories at varying levels of abstraction. The subcategories and categories for the two content areas are presented in Tables 2 and 3. In the end all emerging subcategories and categories were discussed, reflected on and condensed into final subcategories and categories.

**Table 2:** Codes, subcategories and categories in relation to “barriers for weight-loss”

**Codes**	**Subcategories**	**Categories**
Longing for certain food	Difficulties changing food habits	Struggling with self
Dislike the new diet		
Inactivity	Health problems	
Food intolerance		
Stressful life events		
Cheating	Lack of self-control	
Sugar alcoholic		
Cannot control weight		
Food on the brain		
Will I manage?	Insecurity	
Lack of inner strength		
Partner	Social relations	Struggling with implementing the diet
Friends		
Work/travel		
Too little variation	Project-related difficulties	
Costs		
Availability		
Time-consuming		
More support		

**Table 3:** Codes, subcategories and categories in relation to ‘facilitators for weight-loss’

**Codes**	**Subcategories**	**Categories**
Motivated	Clear goals	Striving for self-determination
Refrain from eating		
Partner support	Support from family/friends	Receiving support
Friend support		
Project support	Project-related support	
Project inspiration		

The study has been approved by the Regional Ethics Vetting Board in Umeå. Written informed consent was obtained from all participants.

## Results

The identified categories and subcategories for barriers and facilitators to weight-loss are described below. The codes, subcategories and categories related to barriers to weight-loss are described in Table 2. The results for each category are described below.

### Category: struggling with self

Four subcategories – difficulties in changing food habits, health problems, lack of self-control and insecurity – were identified in relation to Struggling with self.

The women, especially those on the PD diet, said that it was *difficult to change food habits*. They found the first period in the project rewarding due to heavy weight-loss and thus they thought the new diet was acceptable. However, after some time (such as after the first year) the women could long for forbidden food (like cheese, bread, potatoes and wine) and thus found the new diet extremely difficult to keep. They said that the difficulty was to avoid falling back into old habits as time went on, and they longed for excluded food. In the long run, it could be difficult to do without food they liked a lot.

It was even harder if they did not like the food in the diet intervention, for example fruit and vegetables. One participant described how she got very angry when she forced herself to eat the vegetables, which she did not like. But she continued to eat them anyway.

Another barrier to keeping the diet was *health problems*. New or exacerbated health problems could destroy the ambition to lose weight, because these problems could make it more difficult for them to perform daily activities, such as physical activity. For example, one participant broke her leg, and the persistent pain after the operation made it hard for her to be physically active. The increased inactivity made it difficult for her to lose weight and therefore she dropped out of the project. Another participant broke her arm, which made it very difficult to cook. Women with other ailments, such as bile problems or intolerance to e.g., fruit, experienced even more restricted diets after the exclusion of such food products. In addition, women who underwent severe life events such as health problems among partners/relatives said that the stressful situation prevented them from keeping their diet.

*Lack of self-control* was also identified as a major inner obstacle to weight-loss. Following a diet requires high self-control; it was considered too easy to give way to temptation.

The participants talked about cheating, i.e. eating what was not allowed according to the diet regimes. During certain periods of the diet intervention the participants kept a detailed diary of everything they ate, which they received feed-back on from the project leaders. During the food diary week, the women said that they tried their best to keep the diet, but after that week their dietary regime may not have been so perfect any longer.

Cheating was experienced as a major problem for the participants. The project was described as a help to structure their eating in order not to yield to temptation. On the other hand, it was tempting to go back to old, preferred food habits if the project did not lead to the desired weight reduction. The cheating was related to difficulties in finding motivation to change food habits. And through time, this cheating could be the reason why the women dropped out of the project. For those who did not succeed with the project the forbidden food was experienced as a comfort. Consoling oneself by eating was identified as a barrier to weight-loss.

Another reason for cheating was that the women had reached their goals for weight-loss and were able to have a more relaxed relationship to food. The project still expected the participants to keep the diet, despite weight-loss. As one participant said, after a certain time in the project with desirable weight reduction she could lean back, relax and eat some of the food she had given up because of the diet regime. A structural reason for cheating was taking retirement and spending more time at home, thus being closer to food, so to speak.

Being addicted to sweets was experienced as lack of self-control over eating. This sugar addiction seemed to be a major problem for the interviewed women. For example, one participant described herself as a sweet-alcoholic; she just had to eat sweets:

“I cannot buy sweets, which is why I call myself an alcoholic because I’m like them in that they can’t buy themselves a bottle of alcohol. And I have to eat all sweets at once. Nothing else is possible. I wish the sweets were as far away in the shop as possible.”

Another woman talked about a similar addiction to sweets. She thought that her addiction was triggered by bread, or maybe by “something in her head” and also that the problem was inherited, because her daughter was also addicted to sweets:

“and then I want that sweet. And afterwards when I have eaten it I feel sick and I think– hell, why did I eat it … when I know that I should not and that I feel bad afterwards. ”

The women could also experience a more or less total lack of control over eating. As one participant said:

“my weight only increases and increases and never goes down again”.

In her description of herself, she is not in control over her eating or her weight. The sandwich (or a bit later in the interview – the chocolate) “just goes down”.

*Insecurity* was the last subcategory in relation to the category Struggling with self. The women in the diet intervention had long-term experience of unsuccessful dieting and therefore they said they were often insecure about whether or not they would succeed this time. Especially those on the PD diet experienced the food as complicated and therefore a question was: What is right to eat? The women described how the PD diet could bring about too large a change compared to their usual food intake, which added to the feelings of insecurity.

An explanation for not succeeding in weight reduction was lack of inner strength, which also increased the feelings of insecurity. Good appetite in combination with difficulties in resisting eating was described as a bad combination for weight-loss. As one participant said:

“So I tried … but no, I could not mobilise the inner strength which is a pity”.

### Category: struggling with implementing

Struggling with implementing was related to social relations and project-related difficulties. Barriers associated with *social relations* include difficulties in combining dietary changes with being together with the family, friends or workmates. The women said that their partner could be a major obstacle to weight-loss. He could be an obstacle by tempting them with forbidden food, as described by one of the drop-outs. Her new partner brought sweets or baked cakes to her all the time. During their time together he increased his weight a lot. When asked how she managed the weight reduction she answered:

“It’s hard. You can see how he really drowns his food in cream and sugar and then he looks at me like – he knows, he is conscious that this is not good”.

In other cases, the women told us that their partner did not care about their new diet, even though he did not accept eating it himself. The majority of participants said that they lived with a husband/partner who did not cook. He still expected her to make his food in the traditional way; this meant that the wife had to cook two different dishes for every meal:

“My husband had no opinion except that he … wanted to have the usual food with sauce and so on”.

So these women ended up keeping two households – the traditional one for the husband and the new diet for themselves. Only one of the interviewed women talked about the husband/partner sharing the cooking with her. Another obstacle experienced was when their grown-up children with families came to visit them, especially when they expected to be served traditional food rather than the new diet.

Friendships were also identified by the women as a possible obstacle to weight-loss. Having coffee together was described as a central part of social relations, and this coffee always included buns and cakes. The participants described feelings of isolation when they had their fruit while everyone else was having buns and cakes. And being invited to a party always meant being offered sweets, which were found difficult to resist. The women talked about their fear of hurting the hostesses who had made the food and therefore they might eat of everything rather than keep to their diet. Besides, the women were afraid that too much emphasis on their new diet could impose demands on their friends to start dieting too. These social problems were identified as a reason why the participants did not tell others about their new diet. A strategy among the participants was to invite friends to their homes for dinner rather than go to their home, because it was easier to keep the diet when you made the food yourself.

The women who were still working found it easier to keep the diet at work with restricted access to sweets, compared to being at home or invited to friends. On the other hand, it could be very difficult to keep the diet when travelling. Travelling a lot also made it difficult to keep up with the project meetings and with the data collection for the project. Also, night work could disturb any food habits, especially if you were on a new diet.

The participants also identified *project-related difficulties*. A barrier experienced with the PD food was that it was quite restricted. As one participant who had left the study in advance stated:

“There was too little variation for me to keep the diet over a longer time … Everything you longed for, cheese is one example.”

The quotation also indicates that it could be boring to have too little variation in the diet. One participant described how tedious it was to peel one kilogram of shrimps to give 300 grams of shrimps for breakfast every Saturday morning, while her husband could eat anything. It also felt boring to eat fruit when everyone else (at work, at home or with friends) had buns and cakes, or to follow their diet in general, because of lack of variation in the food intake. Being able to eat sweets instead of fruit was seen as adding that little extra to life.

For some of the women, especially those on the PD diet, the new food was very expensive:

“Proteins are very expensive to buy … vegetables are seasonally expensive so that made about 100 euro extra per month which is too much. I do not have the economy”.

The high costs could be a reason for leaving the project. As one participant told us, previously she could grow potatoes without any cost or buy cheap pasta, whereas now she had to buy expensive vegetables and proteins. The costs also depended on their place of living – the costs were especially high if you had no large supermarket close to your home. Participants living in the rural area told us that they had to pay much more for their new diet as compared to urban participants who had large supermarkets nearby.

The PD diet was also found to be more expensive to eat in a restaurant than the traditional diet. The women claimed that proteins together with vegetables cost much more than, for example, a pizza or pasta.

Participation in the project was experienced by some of the women as very time-consuming. This was especially the case for those living outside the main city as well as for those with shift work (including the health care staff) who regularly worked during evenings/nights. For them it was difficult to combine the group meetings and the other project activities (including taking specimens for the project) with their own work, or with long distances to travel from home. The extra time needed for participating in the project together with the extra time needed for preparing the new diet (especially the PD diet) could be an extremely stressful experience and a reason for leaving the project.

Also, some of the participants wanted more support and coaching from the project for their diet change. They said that during the project they had wanted to cook more together as well as to have more group meetings or some kind of contact with the group participants in between the group meetings. Also, one woman expressed a desire for the project to control the amount of food she ate.

Another project-related problem was disappointment about the group to which the women were randomised. The PD group was most popular because the other diet could be experienced as eating the same as their ordinary food.

### Facilitators for weight-loss

The codes, subcategories and categories related to facilitators for weight-loss are described in Table 3. Two main categories were identified – striving for self-determination and receiving support. Overall, the interviews were not as rich in relation to “facilitators” as they were in relation to “barriers for weight-loss”.

### Category: striving for self-determination

One subcategory – *self-determination* – was identified as a facilitator of weight-loss. Those who succeeded in reaching the goals of their weight-loss said that they were very motivated and had clear goals. Also, they easily accepted their new diet. Their self-determination helped them to refrain from undesirable eating. One strategy was to drink less coffee, because coffee drinking was associated with eating buns and cakes. Another strategy was to buy cheese (which many loved) with lower fat and to refrain from buying sweets. The self-control was expressed as “I do not permit myself to eat” or “I forbid myself to eat”. The women could also use their self-control and refrain from dinner invitations to stick to their diet more easily.

### Category: receiving support

Two subcategories were identified in relation to this category – *support from family/friends* and *project-related support*. As stated above in relation to “barriers for weight-loss”, the partner could be an obstacle. But there were also women whose husbands/partners were supportive. One of these women said that her husband thought the new diet was super. But she stopped him from eating her diet because she believed that he was too physically active to feel good with that food. Instead he had to fix his own food.

Friends – just like husbands/partners – could either be supportive or make weight-loss more difficult. When supportive, the friends did not tempt the participants to eat forbidden food. When inviting them for dinner, the friends would cook the diet food too. Also, good friends could be really supportive in helping the participants to keep their diet.

The *project-related support* was described as either direct support or inspiration to diet change. The participants felt that they received support in sticking to their diet from the group meetings as well as from the dieticians and the nurses. The project was also experienced as indirectly supportive since the participants could refer to and use the project in relation to others as an excuse for refraining from certain food.

The project was described as an inspiration to try a new diet and to learn about new ingredients. This was especially true for those who had no financial problems getting hold of the new diet. And some of the participants continued to eat their new diet, especially the breakfast, even after ending the project.

## Discussion

### On the results

In summary, we identified that the women in the dietary intervention experienced two main barriers–struggling with self (related to difficulties in changing food habits, health problems, lack of self-control and insecurity) and struggling with implementing the new diet (related to social relations and project-related difficulties) – and two main facilitators– striving for self-determination (related to having clear goals) and receiving support (from family/friends as well as from the project) – for weight-loss. There was a greater emphasis on barriers than on facilitators.

The balance between barriers and enablers is strongly associated with actual behaviour change according to most theories of behaviour change. The Health Belief Model states that if the perceived barriers to a change in behaviour outweigh the perceived benefits, then the probability of such a change in behaviour will be severely reduced [11]. From Social Cognitive Theory, working with the concept of self-efficacy, one can easily understand a situation where an individual with low self-efficacy for dietary change simultaneously finds a higher frequency of barriers than enablers in daily life when appraising the possibility to make dietary change [12]. In clinical weight-loss programmes (just as in this study) most obese individuals have a history of earlier weight-loss attempts and unsuccessful past experiences concerning weight-loss. A consequence of this is a low level of self-efficacy for achieving weight-loss, especially on a long-term basis. A majority of this group of obese people also report major obstacles or perceived barriers to adherence to weight-loss treatment or healthy eating intervention [13]. The fact that one third of the participants were drop-outs may be one explanation for the strong emphasis on barriers as compared to facilitators.

In addition, qualitative research has shown that obese people often experience their weight in profoundly negative ways due to living in a social context which stigmatises overweight and especially obesity [14]. In order to develop successful interventions, health professionals need to understand individual experiences of being obese and the negative impact of obesity on self-identity. Also, interventions need to be aware that negative individual responses to one’s obesity may be a barrier for weight-loss [14]. In a systematic review, Mold and Forbes [15] examined the experiences of obese persons in relation to their health-care provision and health-care professionals in providing care for obese patients. The review included 30 studies published 1990–2010, showed that obesity has a strong effect on how obese individuals view themselves. Obese patients have often in everyday life at numerous occasions been subjected to weight-based stigma and discrimination, which reduces their status, increases their self-blame and their negative self-image as well as their sense of powerlessness. The stigmatisation is also related to negative psychobehavioural responses such as maladaptation and low self-esteem, which may impede the ability to adopt positive behaviours in order to overcome barriers for weight-loss [15]. Experiences of stigmatisation also have effect on how they access and interact with health-care providers. The review suggests that there exist many forms of discriminatory practices among health-care providers towards obese patients [15].

As there is a limited amount of qualitative research about personal experiences of barriers and facilitators to weight-loss in diet interventions, our findings are novel within the medical literature. A few qualitative studies within the field of diet interventions have been performed. One article about a US study made a qualitative assessment of barriers and facilitators to achieving behaviour goals among obese inner-city adolescents in a weight management programme [16]. Because of the different context and ages the results of that study are difficult to compare with ours. Barriers to reaching physical activity goals among girls in this US study included unsafe neighbourhoods and a negative body image. Maintaining unrealistic behaviour and weight goals hindered weight-loss in both men and women. What was similar to our results was the positive impact of coaching within the intervention. It helped the teens feel more successful in the goal-setting process and addressed issues related to their disruptive environments.

When comparing our results with an article based on a qualitative study of Arab women at risk of type 2 diabetes [17] we find not only similarities (related to the importance of social support) but also contextual differences such as socio-cultural norms that restrict outdoor physical activities among the Arab women.

When comparing our findings with quantitative population-based studies we find a similar barrier related to high costs of healthy food in an Australian study of young women [18]. Lack of motivation and lack of time were additional major barriers in that study. The selection of motivated participants in our study could explain the different findings.

A qualitative study of US college students [19] found similar barriers to weight management among the young men and women to those we found in our selected group of older Swedish women: intrapersonal (such as temptation and lack of discipline); interpersonal (social situations); and environmental (e.g., time constraints, ready access to unhealthy food). Facilitators similar to those in our study were also identified among the young men and women in the US study–intrapersonal (e.g., regulating food intake, being physically active) and interpersonal (social support) [19]. While our study focused on an intervention, the US study focused on creating a supportive environment for physical activity. As in our study, more barriers than enablers were given, indicating that the participants in these studies are more sensitive to the barriers than the enablers of weight management.

Another US study [20] – in this case quantitative – of patients and providers in primary care showed that the patients and GPs had very different views of barriers and facilitators to weight-loss. As in our study, lack of self-control and need for support to stay on a diet was emphasised by the GPs while the patients wanted to manage their weight problems on their own.

Lack of self-control in relation to sweets was described by some participants as sugar addiction and equated with alcoholism. This concept has received much attention in popular lifestyle magazines, but the scientific evidence is contradictory. Animal models find that rats under certain circumstances can become dependent on sugar [21]. However, sugar as a substance causing physical addiction in humans is rejected in a review study [22].

Our study included women, and a relevant question is whether or not our results are transferable to men. One major topic was that the women in our study did almost all the cooking. Because men seldom cook, they are dependent on their wives for getting the new diet. Research has shown that if the husband contracts diabetes, the whole family changes their diet, but if the wife gets diabetes, the family wants to continue with their traditional food so she cooks traditional food for herself and diet-restricted food for her husband [23]. The explanation given was that the husbands would not accept a new diet if they do not need it themselves.

Thus, a gender-relational approach [24] – in which men’s and women’s relations and interactions with each other are analysed in different settings, such as family life – seems to be reasonable to understand our findings. With this theoretical framework, the relations between the women and their partners/husbands can be understood from a societal perspective of men’s superiority and power in relation to women. Women – even in a gender-equal society like Sweden – perform most of the domestic work [25], which in the case of the women in our study meant that they were the food providers of the family. So when their husbands refused to eat their new diet, they had to cook two meals. That in itself could be a major obstacle to weight-loss. Thus, the power dynamics within the couple regarding food preparation put women in a subordinate position vis-à-vis their husbands. A significant example of this subordinate position was the stories from women who were tempted by their partners/husbands to eat forbidden food. Thus, a gender-relational approach interprets our findings as showing that women’s subordinate position in the couple relationship constituted a major barrier to weight-loss.

This interpretation is in line with a qualitative study focusing on food practices among Finnish men in which the men described cooking as optional or exceptional [26]. Their masculinities were seen as constructed from a rejection of feminine ideals such as everyday cooking. Another study, with an approach based on a relational theory of gender, focused on diet change among men with prostate cancer [27]. The study emphasised how both wives and husbands mutually limited men’s engagement in cooking and diet while reinforcing women’s traditional femininities in nurturing their husbands. The same interpretation could be used in relation to our findings about the woman who described how she stopped her husband from adopting her diet because of her considerations about his need for energy. In spite of unequal power relations associated with women’s responsibility for the domestic sphere, the women might construct themselves as wanting to have the main control over traditional feminine duties such as cooking [28]. Overall, there is a lack of research from a gender-relational approach about the impact on health of the gender division of cooking [27,29].

Our findings can also be compared to one of the few studies on men, in this case a qualitative study of Danish younger working-class men, motivated for weight-loss [30]. Lack of motivation together with a negative perception of the slimming diet were the main barriers. It is hard to know what made the result so different from our study but probably the combination of age, gender and class as well as (perhaps most importantly) the selection of very well-motivated women into our study.

### On the methods

The trustworthiness of our results needs to be considered [9]. Trustworthiness comprises the four concepts credibility, confirmability, dependability and transferability and we discuss them according to Lincoln and Guba [31,32]. The credibility or truth value, deals with whether our findings were built on faithful conditions [31,32]. The interview guide was discussed and decided upon in the group of authors who analysed the interviews (AH, AFW, CA), all three experienced qualitative researchers from different disciplines (triangulation between researchers) [31]. The interviews were performed with open questions by a skilled interviewer, who did not take part in the analysis. After each interview, the interviewer reflected upon and discussed any adjustments in the interview guide together with the authors. Overall, the interviews were rich and relatively long. The text was analysed by three of the authors (AH, AFW, CA). The process has been open and transparent and the analyses have been carefully examined and also discussed in depth by the authors with the interviewer taking part, as well as in various seminar groups (peer debriefing) [31]. Confirmability deals with to what extent the findings were affected by personal interests and biases [31,32]. The interviewer along with the authors who analysed the data did not take part in the RCT-intervention and thus could bring an outsider perspective on the data. Dependability deals with how stable the data are over time and conditions during the study [31]. Women from both dietary groups as well as drop-outs from the whole 2 -year study period were invited to participate in the interviews. In the Result Section, examples of quotes and the whole coding process are provided in Tables 1 and 2 (audit trail) [31,32]. The results have been thoroughly discussed within the whole group of authors as well as with other groups of researchers who found the result to be credible. Thus, we consider our results trustworthy.

The transferability of our findings needs to be reflected upon in relation to contextualisation and sampling procedures [31,32]. Our findings are strongly contextualised within the frame of the 2-year RCT diet intervention among motivated middle-aged women in Northern Sweden. Our inclusion of appropriate quotations also enhances transferability. It can be questioned whether the text from 12 interviews was enough. The interviewed women were strategically selected regarding their social background, age, civil status and living conditions. The interviews were rich and gave varied views on the topics of this paper. Therefore, we believe that our results can be transferred to similar groups and contexts to those represented by the women in our study.

The main limitation of our study was the homogeneous sample of middle-aged white women in Northern Sweden in a very specific diet intervention. In spite of that, we found not only similarities but also differences in relation to the main findings. Even so, more research is needed about experiences of weight-loss interventions in other contexts.

## Conclusions

Surprisingly few studies have analysed barriers and facilitators to weight-loss. It is important to also include drop-outs from diet interventions in order to fully understand barriers to weight-loss. A gender-relational approach can bring new insights into understanding experiences of barriers to weight-loss.

## Competing interests

The authors declare that they have no competing interests.

## Authors’ contributions

AH, AFW, BL and CA designed the qualitative study, while CL and BL designed the diet intervention. AFW and CA performed interviews, which were analysed by AH, AFW and CA. AH wrote the manuscript which all co-authors commented upon. All authors read and approved the final manuscript.

## Pre-publication history

The pre-publication history for this paper can be accessed here:

http://www.biomedcentral.com/1472-6874/14/59/prepub
